# Neurodevelopmental Genetic Diseases Associated With Microdeletions and Microduplications of Chromosome 17p13.3

**DOI:** 10.3389/fgene.2018.00080

**Published:** 2018-03-23

**Authors:** Sara M. Blazejewski, Sarah A. Bennison, Trevor H. Smith, Kazuhito Toyo-oka

**Affiliations:** Department of Neurobiology and Anatomy, Drexel University College of Medicine, Philadelphia, PA, United States

**Keywords:** 17p13.3, microdeletion, microduplication, lissencephaly, autism spectrum disorder, neurodevelopmental disorder, CRISPR, next generation sequence

## Abstract

Chromosome 17p13.3 is a region of genomic instability that is linked to different rare neurodevelopmental genetic diseases, depending on whether a deletion or duplication of the region has occurred. Chromosome microdeletions within 17p13.3 can result in either isolated lissencephaly sequence (ILS) or Miller-Dieker syndrome (MDS). Both conditions are associated with a smooth cerebral cortex, or lissencephaly, which leads to developmental delay, intellectual disability, and seizures. However, patients with MDS have larger deletions than patients with ILS, resulting in additional symptoms such as poor muscle tone, congenital anomalies, abnormal spasticity, and craniofacial dysmorphisms. In contrast to microdeletions in 17p13.3, recent studies have attracted considerable attention to a condition known as a 17p13.3 microduplication syndrome. Depending on the genes involved in their microduplication, patients with 17p13.3 microduplication syndrome may be categorized into either class I or class II. Individuals in class I have microduplications of the *YWHAE* gene encoding 14-3-3ε, as well as other genes in the region. However, the *PAFAH1B1* gene encoding LIS1 is never duplicated in these patients. Class I microduplications generally result in learning disabilities, autism, and developmental delays, among other disorders. Individuals in class II always have microduplications of the *PAFAH1B1* gene, which may include *YWHAE* and other genetic microduplications. Class II microduplications generally result in smaller body size, developmental delays, microcephaly, and other brain malformations. Here, we review the phenotypes associated with copy number variations (CNVs) of chromosome 17p13.3 and detail their developmental connection to particular microdeletions or microduplications. We also focus on existing single and double knockout mouse models that have been used to study human phenotypes, since the highly limited number of patients makes a study of these conditions difficult in humans. These models are also crucial for the study of brain development at a mechanistic level since this cannot be accomplished in humans. Finally, we emphasize the usefulness of the CRISPR/Cas9 system and next generation sequencing in the study of neurodevelopmental diseases.

## General introduction

Chromosome 17 boasts the third highest density of segmental duplications among human chromosomes and has the second-highest gene density. More than 23% of the short arm of chromosome 17 consists of low-copy repeats (LCRs), creating the opportunity for non-allelic homologous recombination (NAHR) to occur (Komoike et al., 2010; Shimojima et al., 2010). This differs from allelic homologous recombination (AHR), which serves to separate haplotypes and results in the interchange of homologous sequences to contribute to non-pathologic genetic variation. NAHR results in the recombination of sequences on different chromosomes, different positions of homologous chromosomes, or between two sequences on the same chromosome. This can lead to deletions and duplications (Clancy and Shaw, 2008). The high density of LCRs found within chromosome 17p13.3 makes it a “recombination hotspot.” LCRs are made up of the same or very similar repetitive sequences and are ~95% identical (Stankiewicz et al., 2003). Many deletion breakpoints have been identified within LCRs (Stankiewicz et al., 2003). All of these factors contribute to the association of chromosome 17p13.3 with rare genetic diseases caused by haploinsufficiency or duplication.

The instability seen in chromosome 17 contributes to the development of a wide variety of diseases including morphological brain disorders, mental illnesses, epilepsy, and tumors (De Smaele et al., 2004; Shimojima et al., 2010; Schnaiter and Stilgenbauer, 2013; Gazzellone et al., 2014). In this review, the four major phenotypes we will discuss are Miller-Dieker syndrome (MDS), isolated lissencephaly sequence (ILS), class I 17p13.3 microduplication syndrome, and class II 17p13.3 microduplication syndrome. The *CRK, PAFAH1B1*, and *YWHAE* genes located at chromosome 17p13.3 all have crucial roles in neuronal migration and contribute to each of these genetic disorders when microdeletions or microduplications arise. MDS and ILS are associated with 17p13.3 microdeletions. Earl Walker first described cases of lissencephaly in 1942 in reference to the “smooth brain” observed. Miller and Dieker contributed to the identification of MDS in 1963 and 1969 respectively, resulting in the nomenclature of the disease (Walker, 1942; Miller, 1963; Pilz and Quarrell, 1996). MDS results from contiguous gene deletion within 17p13.3 and mainly features cerebral agyria (absence of gyri), cerebral pachygyria (broad gyri), craniofacial deformities, and seizures (Barros Fontes et al., 2017). ILS phenotypes lack the craniofacial deformities and the most severe grade of lissencephaly only observed in MDS phenotypes. These features of MDS patients result from larger deletions within chromosome 17p13.3 as compared to ILS patients. MDS is caused by microdeletions containing *PAFAH1B1* and *YWHAE*, at minimum, while ILS can result from heterozygous mutation or deletion of *PAFAH1B1* (Dobyns et al., 1993; Reiner et al., 1993). The thickening and simplification of cortical layers associated with lissencephaly seen in both ILS and MDS are due to a defect of neural migration during cortical development. This results in a variety of defects, including mental retardation (Dobyns et al., 1993; Cardoso et al., 2003).

Patients with microduplications of 17p13.3 were first reported in 2009, and this condition is now referred to as 17p13.3 microduplication syndrome (Bi et al., 2009; Roos et al., 2009). Although there are currently only about 40 reported cases, the number of 17p13.3 microduplication syndrome patients has been increasing. The affected can be categorized into two classes based on the size of chromosome duplication (Bruno et al., 2010). Patients with class I microduplications never have *PAFAH1B1* duplications. They typically display autistic and other behavioral symptoms, delay in speech and motor abilities, craniofacial deformities, hand and foot deformities, and postnatal overgrowth (Bruno et al., 2010). In contrast, *PAFAH1B1* is always duplicated in patients with class II, who may also have duplications of *CRK* and *YWHAE* (Bruno et al., 2010). Microduplications in class II result in psychomotor and developmental delay, as well as hypotonia (Bruno et al., 2010). Microdeletions and microduplications around the MDS critical region, which is the region of chromosome 17 spanning from *PAFAH1B1* to *YWHAE*, have distinct phenotypes. Even so, both share similarities and affect the same genes (Table 1).

**Table 1:** Comparison of major phenotypes and genotypes associated with microdeletions and microduplications.

	**Microdeletions**		**Microduplications**	
	**ILS**	**MDS**	**Class I**	**Class II**
Major phenotypes	Lissencephaly (grade 2–4) Epilepsy	Lissencephaly (grade 1) Epilepsy Craniofacial dysmorphisms Congenital anomalies	Learning disabilities Autism spectrum disorder Elevated growth factors Dysmorphic facial features Developmental delay Behavioral problems (severe)	Developmental delay Psychomotor delay hypotonia Mild brain malformations Behavioral problems (mild)
Major genotypes	Deletion of *PAFAH1B1*	Deletion of *PAFAH1B1, YWHAE*, and/or *CRK*	Duplication of *YWHAE* and/or *CRK*	Duplication of *PAFAH1B1*, and/or *YWHAE*, and/or *CRK*
References	Cardoso et al., 2003; Nagamani et al., 2009; Takahashi et al., 2015		Bi et al., 2009; Bruno et al., 2010; Curry et al., 2013	

**Figure 1:**
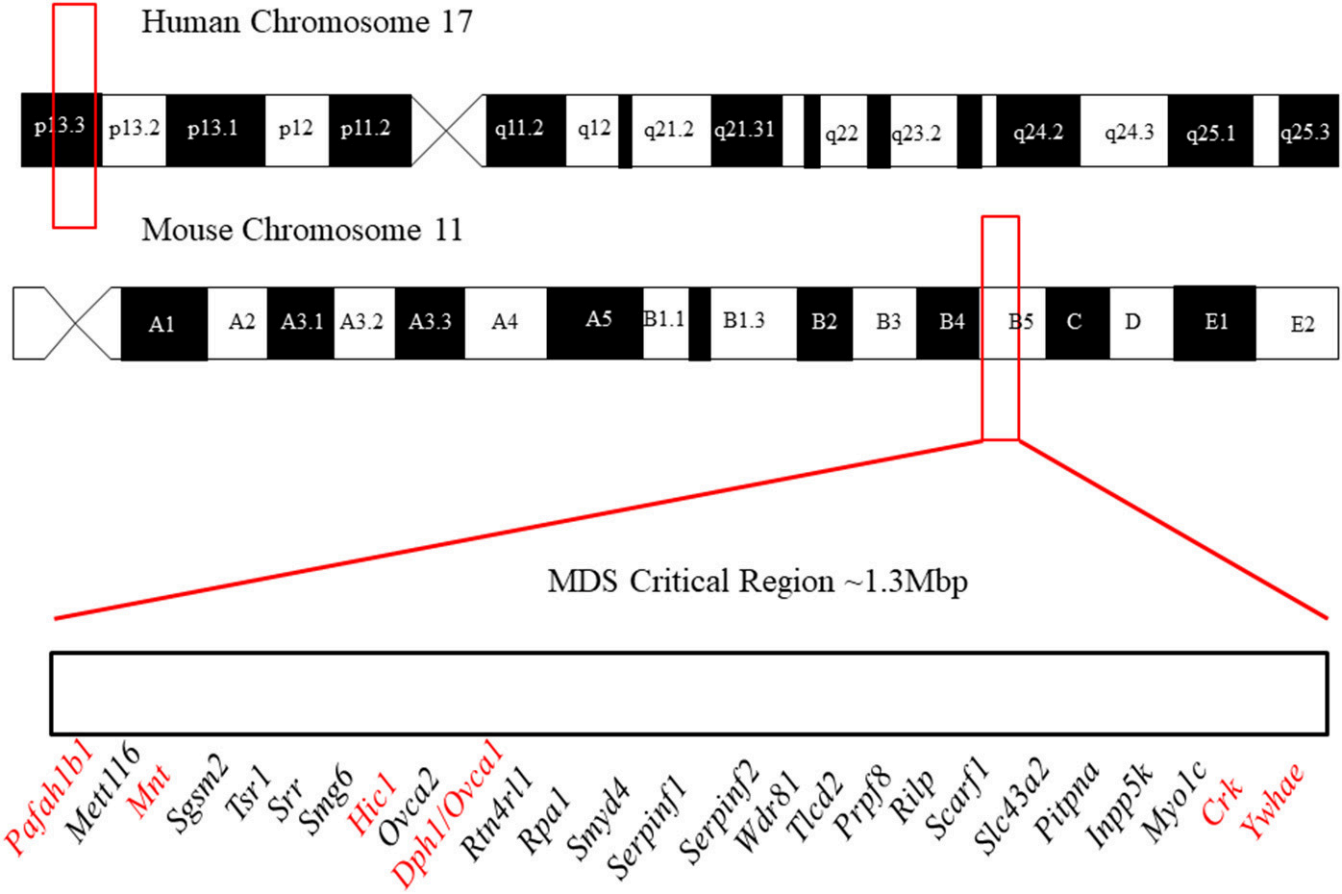
Outline of the location of the MDS critical region in humans and mice. In humans, it lies in region 17p13.3 and in mice it lies on chromosome 11 in region B5. The gene order of the MDS critical region in mice is defined above. Figure not to scale. The order and position of genes were obtained from the Human Genome Project Ensembl Database (release 90, accessed on 10/23/2017).

It is difficult to study these conditions with human patients due to the small number of affected individuals. Fortunately, the areas of clinical interest within the MDS region are largely conserved between the human and mouse genome (Figure 1). The short arm of chromosome 17 in humans coincides approximately with the center of chromosome 11 in mice and the 26 human genes (Table 2) known in 17p13.3 have homologs in the mouse chromosome. The genes in the MDS critical region are in the same order in humans and mice; however, some neighboring genes are in a different order. These genetic similarities and the parallels between human and mouse development make the mouse model an excellent analog (Yingling et al., 2003). Previous studies have used knockout and transgenic mice to study genes within the MDS critical region. There are currently some single and double knockout mice available, which will be discussed. However, mouse models for the complete deletion or duplication of chromosome 17p13.3 have yet to be developed. In this review, we discuss microdeletions and microduplications of chromosome 17p13.3 with a focus on patient phenotypes, phenotypes seen in single and double knockout and transgenic mice, and limitations on current studies. We also propose a combined CRISPR/Cas9-Cre-loxP approach to create mouse models with microdeletions and microduplications of the complete chromosome 17p13.3 region, which may advance current studies by providing a more clinically relevant model. We then discuss the potential for applied analysis of next generation sequencing to improve identification of copy number variants.

**Table 2:** Summary of genes involved in microdeletions and microduplications of human chromosome 17p13.3.

**Gene**	**Functional relevance**	**References**
BHLHA9^*^	Encodes a protein from the basic helix-loop-helix family; transcription factor involved in limb development	Klopocki et al., 2012
*YWHAE*	Encodes protein 14-3-3ε which is a phosphoserine/threonine-binding protein and a critical regulator of neurogenesis and neuronal differentiation	Cornell et al., 2016
*CRK*	Encodes a signaling protein downstream of Reelin; functions in cell proliferation, differentiation, migration, axonal growth	Huang et al., 2004; Matsuki et al., 2008; Park and Curran, 2008; Barros Fontes et al., 2017
*MYO1C*	Encodes a member of the unconventional myosin family (myosin class 1); actin-based molecular motor with ATPase activity; nuclear isoform associates with RNA polymerase I and II to initiate transcription	Desh et al., 2014; Sarshad et al., 2014; Arif et al., 2016
*INPP5K*	Encodes a protein that is a potential negative regulator of the actin cytoskeleton	Rahman et al., 2011
*PITPNA*	Encodes a protein member of a family of lipid-binding proteins that transfer molecules of phosphatidylinositol/choline between membrane surfaces; implicated in phospholipase C signaling; implicated in production of phosphatidylinositol 3,4,5-triphosphate	Kular et al., 1997; Morgan et al., 2004
*SLC43A2*	Encodes a member of SLC43 (L-amino acid transporter 3) transporter protein family; mediates transport of L-isomers of neutral amino acids in a pH-, sodium-, and chloride- independent manner; implicated in the transfer of amino acids across the placenta to the fetus	Bodoy et al., 2005, 2013
*SCARF1*	Encodes scavenger receptor protein that is expressed in endothelial cells; regulates uptake of chemically-modified low-density lipoproteins	Dhaliwal and Steinbrecher, 1999; Ishii et al., 2007
*RILP*	Encodes a lysosomal protein; potentially a downstream regulator of RAB7; RAB7 and RILP potentially interact to regulate endocytic traffic; potential regulator of lysosomal morphology	Cantalupo et al., 2001; Jordens et al., 2001; Harrison et al., 2003; van der Kant et al., 2013
*PRPF8*	Encodes a protein that is a part of the U2- and U12- dependent spliceosomal machinery; essential for step II of pre-mRNA splicing	Grainger and Beggs, 2005; Boesler et al., 2015; Wickramasinghe et al., 2015
*TLCD2*	Encodes TLC domain containing two proteins; unknown function	Zody et al., 2006
*WDR81*	Encodes a multi-domain transmembrane protein; predominantly expressed in brain; potential role in endolysosomal trafficking	Liu et al., 2017; Shaheen et al., 2017
*SERPINF2*	Encodes a member of the serpin family of serine protease inhibitors; major inhibitor of plasmin, which degrades fibrin and various other proteins	Abdul et al., 2016
*SERPINF1*	Encodes a member of the serpin family (no protease inhibitory activity, secreted protein); strongly inhibits angiogenesis, neurotrophic factor	Johnen et al., 2015
*SMYD4*	Encodes a protein with a SET domain, which often has lysine methyltransferase activity for histone modification; potential tumor suppressor gene	Hu et al., 2009
*RPA1*	Encodes the largest subunit of the heterotrimeric Replication Protein A (RPA) complex; role in recruiting molecules to DNA	Dubois et al., 2017; Zhang et al., 2017
*RTN4RL1*	Encodes a protein with a GPI-anchored domain and LRR motif; also known as Nogo receptor 3 (NgR3), a paralog of NgR1	Zhang et al., 2011
*DPH1/OVCA1*	Encodes an enzyme involved in the biosynthesis of diphthamide; defects associated with autosomal recessive intellectual and craniofacial dysmorphisms	Loucks et al., 2015
*OVCA2*	Encodes a protein that is related to a variety of α-β hydrolases including esterases, lipases, and other enzymes; highly conserved gene	Prowse et al., 2002
*HIC1*	Tumor repressor gene, cell cycle regulator	Kumar, 2014; Janeckova et al., 2015; Hao et al., 2017
*SMG6*	Encodes a component of the telomerase ribonucleoprotein complex; role in nonsense-mediated mRNA decay (NMD) pathway	Chakrabarti et al., 2014; Nicholson et al., 2014; Schmidt et al., 2015; Colombo et al., 2017; Ottens et al., 2017
*SRR*	Encodes serine racemase, which produces the NMDA co-agonist D-serine that is implicated in Alzheimer's disease, ALS, ischemic brain damage, and schizophrenia	Nelson et al., 2017
*TSR1*	Encodes a serine-arginine-rich protein; splicing factor, affects mRNA stability and rRNA processing	Gupta et al., 2014
*SGSM2*	Encodes a GTPase activator; regulates enzyme trafficking	Marubashi et al., 2016
*MNT*	Encodes a protein in the Myc/Max/Mad network that has a basic-Helix-Loop-Helix-zipper domain; represses transcription	Link and Hurlin, 2015
*METTL16*	Encodes a methyltransferase; required for MAT2A splicing (MAT2A encodes the SAM synthetase expressed in most cells)	Pendleton et al., 2017
*PAFAH1B1*	Encodes non-catalytic α subunit of the intracellular Ib isoform of platelet-activating factor acetylhydrolase, which catalyzes the removal of an acetyl group on platelet-activating factor; deletion results in classical lissencephaly	Nagamani et al., 2009

Genes are listed in the approximate order they are found in the sequence. NCBI Gene Database: Gene [Internet]. Bethesda (MD): National Library of Medicine (US), National Center for Biotechnology Information; 2004—[cited 2017 10 19] (Available from: https://www.ncbi.nlm.nih.gov/gene/).

* *Located within chromosome 17p13.3, but immediately outside the MDS critical region*.

## Chromosome 17p13.3 deletions

### Introduction

Deletion of the 17p13.3 chromosome region results in an array of phenotypes in humans, depending on the severity of the deletion. Well-characterized phenotypes that may result from such a deletion include isolated lissencephaly sequence (ILS) and Miller-Dieker syndrome (MDS) (Ledbetter et al., 1992; Cardoso et al., 2003; Bruno et al., 2010; Barros Fontes et al., 2017). A region of clinical interest within chromosome 17p13.3 is book-ended by two genes: *PAFAH1B1* and *YWHAE*. Deletion of *PAFAH1B1* results in classical lissencephaly (LIS), while larger deletions between *PAFAH1B1* and *YWHAE*, or in other words the MDS critical region, result in MDS (Cardoso et al., 2003; Nagamani et al., 2009). Deletions within the 17p13.3 region are associated with features such as intellectual disability, craniofacial defects, epilepsy, and a few rare symptoms.

Although both ILS and MDS are associated with LIS, they are different conditions. Patients with ILS lack many of the facial dysmorphic features seen with MDS (Ledbetter et al., 1992; Cardoso et al., 2003; Kim et al., 2011). Other clinical features not found in ILS patients, such as cardiac defects, cystic kidney, and polydactyly, may also occur in MDS patients (Cardoso et al., 2003). MDS is associated with the most severe grade of LIS (grade 1), while individuals with ILS have less severe LIS (grades 2–4). The difference between the MDS and ILS phenotypes results from the deletion of specific genes within the 17p13.3 region. Deletion of *PAFAH1B1* along with *YWHAE* and/or *CRK* may cause the more severe grade of LIS seen in MDS patients (Cardoso et al., 2003). *YWHAE* encodes 14-3-3ε, while *CRK* encodes a signaling protein that functions downstream of Reelin and is involved in cell proliferation, differentiation, migration, and axonal growth (Huang et al., 2004; Matsuki et al., 2008; Park and Curran, 2008; Barros Fontes et al., 2017).

Previously, MDS was incorrectly thought to be an autosomal recessive disorder because it was occasionally reported in individuals within the same family (Ledbetter et al., 1989). It has been shown that MDS results from a *de novo* microdeletion of chromosome 17p13.3 that may have a paternal bias in origin (Ledbetter et al., 1989). It is generally accepted that MDS is a contiguous-gene deletion syndrome, meaning that it results from the deletion of multiple genes found in close proximity to each other on the chromosome. This was challenged by a study involving five MDS patients using polymerase chain reaction (PCR) and fluorescence *in situ* hybridization (FISH) to check for microdeletions in chromosome 17p13.3. A deletion was only found in three of the patients. Because all five patients had nearly identical clinical symptoms, the results suggest that MDS is not a contiguous gene-deletion syndrome (Kohler et al., 1995). These data contradict the widely accepted belief that MDS is a contiguous gene-deletion syndrome, but should not be ignored. It is estimated that there is a visible or submicroscopic deletion of chromosome 17p13.3 in 90% of MDS patients, but the cause of the other 10% of cases is unclear (Ledbetter et al., 1992).

ILS may occur when a smaller deletion of chromosome 17p13.3 occurs. About 60% of ILS cases are associated with a deletion or intragenic variation of *PAFAH1B1* (Takahashi et al., 2015). One study found 17p13.3 deletions in only 6 out of 45 ILS patients, which suggests that intragenic variations such as point mutations may account for the majority of cases (Ledbetter et al., 1992).

Deletions of chromosome 17p13.3 result in LIS, which is associated with mental retardation and epilepsy seen in both ILS and MDS patients. Individuals with deletions of *PAFAH1B1* often have ILS, while individuals with larger deletions of the MDS critical region have MDS. More severe lissencephaly and craniofacial dysmorphic features are seen in MDS patients. The relationship between critical genes and human phenotypes will be described next.

### Phenotypes and genotypes in human patients

Almost all of our knowledge about the various phenotypes seen in patients with MDS comes from clinical case studies. Lissencephaly and craniofacial dysmorphism are well-characterized features of MDS. Epilepsy is another major feature of MDS, though this is also seen in ILS. Deletions of certain genes are strongly associated with some phenotypes we will describe.

### Phenotypes: lissencephaly

Classical lissencephaly (LIS) largely results from arrest or defect of neuronal migration occurring between 10 and 14 weeks of gestation, causing abnormal cortical layering and a “smooth brain”, meaning that patients with LIS have an absence or reduction of gyri and sulci in the cerebral cortex (Ledbetter et al., 1992; Wynshaw-Boris and Gambello, 2001; Wynshaw-Boris et al., 2010; Moon and Wynshaw-Boris, 2013). LIS is typically associated with mental retardation, intractable epilepsy, spasticity, and reduced longevity (Dobyns et al., 1993). The severity of LIS ranges from agyria (LIS grade 1), mixed agyria-pachygyria (LIS grades 2 and 3), and pachygyria (LIS grade 4). Clinical symptoms correlate with the degree of agyria (Dobyns et al., 1999). LIS may also be caused by variations of the *DCX* gene on chromosome Xq23, but these cases may be distinguished from those associated with *PAFAH1B1* because of differences in the gyral patterns and more common hypoplasia of the cerebellar vermis in individuals with *DCX* variations (Dobyns et al., 1999; Takahashi et al., 2015; Ayanlaja et al., 2017). Variations of *PAFAH1B1* are linked to a posterior-to-anterior gradient of lissencephaly, while mutations of *DCX* are associated with an anterior-to-posterior gradient (Dobyns et al., 1999). Additionally, *DCX* mutations are associated with lissencephaly in males and a subcortical band heterotopia pattern in females, since *DCX* is located on the X chromosome (Gleeson et al., 1998; Poduri et al., 2013).

It has been established that LIS results from a defect in radial migration. However, previous reports indicate the involvement of interneurons, which have been shown to migrate non-radially (Pancoast et al., 2005). LIS is associated with mental retardation and epilepsy, which can often be linked to interneuron defects (Pancoast et al., 2005). A study involving two fetuses and two children with MDS found a non-radial migration defect in the calretinin-expressing interneuron subpopulation (Pancoast et al., 2005). Pancoast et al. hypothesizes that the clinically distinct forms of LIS have differences in interneurons, which may be useful for diagnosis and treatment once better understood (Pancoast et al., 2005). The idea that the migration of different cell types may be defective in different forms of LIS is supported by work from Marcorelles et al. (2010). A defect in tangential migration of GABAergic neurons has been associated with MDS, while defects in other aspects of tangential migration were reported for two other types of LIS (Marcorelles et al., 2010). Clearly identifying patterns in structural differences, like variations of gyral patterns and migration patterns, will aid in distinguishing between the types of LIS and could have some clinical applications.

While abnormal neuronal migration has been shown to contribute significantly to the lissencephaly phenotype, defects in neurogenesis may also play a role. LIS1, which is encoded by *PAFAH1B1*, influences cell proliferation at various neurodevelopmental stages (Tsai et al., 2005; Bi et al., 2009; Pramparo et al., 2010, 2011; Reiner and Sapir, 2013). A study where LIS1 levels were reduced throughout cortical development showed that LIS1 is involved in generating neuroblasts and postmitotic neurons and also has an influence on cell survival (Gambello et al., 2003). Lissencephaly in patients is analogous to the cortical disorganization observed in LIS1 deficient mice, which can be attributed to a combination of migration defects and LIS1-mediated reduction of cell numbers in the ventricular zone via modulation of cell proliferation and neuroblast survival (Gambello et al., 2003). It seems logical that defects in neurogenesis would impact subsequent stages of neurodevelopment since neurogenesis precedes and could potentially influence many events in development. Additionally, an increased time period in which neurogenesis occurs in a species has been evolutionarily correlated with the degree of convolution observed in the cortex. For example, neurogenesis is three times longer in ferrets and ten times longer in primates, as compared with rodents (Poluch and Juliano, 2015). A longer period of neurogenesis is also correlated with a slower depletion of the cortical progenitor pool (Kornack and Rakic, 1998; Calegari et al., 2005). This provides further support for neurogenesis as a contributing factor in the LIS phenotype, in addition to neuronal migration arrests or defects.

### Phenotypes: epilepsy

Lissencephaly is closely linked with epilepsy. Since LIS occurs in patients with both ILS and MDS, it is a symptom common to both conditions. As with lissencephaly, *YWHAE, CRK*, and *PAFAH1B1* have complicated roles in epilepsy. Two patients with deletions of *YWHAE* and *CRK*, but not *PAFAH1B1*, were described as having macrocephaly, epilepsy, and non-specific changes in white matter, among other clinical features (Tenney et al., 2011). Tenney et al. suggests that deletions of *YWHAE* and/or *CRK* indicate that a patient should be monitored carefully for seizure development (Tenney et al., 2011). Shimojima et al. supports this with similar findings regarding one of the three patients involved in the study (Shimojima et al., 2010). A different patient involved in this case study had a partial deletion of *PAFAH1B1* resulting in isolated grade 3 lissencephaly and epilepsy (Shimojima et al., 2010). Thus, *YWHAE, CRK*, and *PAFAH1B1* are critical genes involved in epilepsy.

### Phenotypes: craniofacial dysmorphisms

Individuals with MDS have a characteristic facial appearance including tall square forehead, flattened midface, low-set posteriorly rotated ear, short upturned nose, and prominent lateral nasal folds (Cardoso et al., 2003; Nagamani et al., 2009; Bruno et al., 2010; Yu et al., 2014). One study involved a patient with a 284 kb deletion within the MDS critical region where *CRK* and *MYO1C* were deleted, but not *YWHAE* (Ostergaard et al., 2012). The patient had mental retardation in addition to slight facial and limb abnormalities, suggesting that *CRK*, but not *YWHAE*, may have a role in craniofacial defects and limb malformations (Ostergaard et al., 2012).

Additionally, deletion of the *HIC1* and *OVCA1* genes has a role in producing these craniofacial dysmorphisms (Barros Fontes et al., 2017). A study involving 19 patients with a 17p13.3 microdeletion reported that 13 of those individuals had haploinsufficiency of *HIC1* and *OVCA1* (Tenney et al., 2011). Cleft palate was observed in 4 of these patients, while craniofacial dysmorphisms were found in 11. These data implicate *HIC1* and *OVCA1* in craniofacial defects and provide evidence for their role in palatogenesis (Barros Fontes et al., 2017). Cardoso et al. identified a 400 kb critical region that differentiates ILS from MDS (Cardoso et al., 2003). Since craniofacial dysmorphisms are unique to MDS, the study of this critical region could help identify more genes involved in these characteristic craniofacial defects.

### Rare and miscellaneous characteristics of MDS

Approximately 90% of MDS patients have a visible or submicroscopic deletion of chromosome 17p13.3 (Ledbetter et al., 1992). About 1 in 50,000–100,000 patients has a ring chromosome disorder (Kim et al., 2014). It is possible for deletions to occur during the formation of ring chromosomes, since they result from the fusion of the short and long arms (Kim et al., 2014). Ring chromosomes have been linked to birth defects and mental disabilities (Kim et al., 2014). Several individuals have been described as having a ring chromosome 17 (Ono et al., 1974; Qazi et al., 1979; Chudley et al., 1982; Sharief et al., 1991). Of those patients, a few have had clinical features consistent with MDS (Sharief et al., 1991). One case report described a MDS patient who had 46 chromosomes with a ring structure taking the place of one of the chromosomes 17. There was also a deletion equivalent to what is typically seen in MDS (Sharief et al., 1991). Recent developments may eventually lead to therapeutics for such individuals. When fibroblast cells derived from patients with ring chromosomes are reprogrammed into induced pluripotent stem cells (iPSCs), the cells lose the ring chromosome and duplicate the wild-type homolog through compensatory uniparental disomy (UPD) (Bershteyn et al., 2014). These results could have important clinical implications and may eventually lead to the development of an approach to correct large-scale chromosomal aberrations (Kim et al., 2017).

While lissencephaly, craniofacial dysmorphism, and epilepsy are seen in the vast majority of MDS patients, there are also some less common and minor symptoms associated with MDS. An example is spinal manifestations. A 6-month-old infant with MDS was reported to have a tethered spinal cord, with the conus medullaris terminating abnormally low at the upper level of L4 (Hsieh et al., 2013). An inflamed lumbosacral sinus dermal tract was also described (Hsieh et al., 2013). The low incidence of MDS makes it difficult to determine if the rare and minor symptoms reported from an individual case study are a direct result of MDS or if they are unrelated. Mouse models with gene mutations will help overcome this difficulty and provide a better understanding of the relationship between specific genes and minor symptoms.

### Mouse models

The region of chromosome 17p13.3 that is frequently deleted in patients with MDS is homologous to a region within the mouse chromosome 11B5. *Mnt, Hic1*, and *Ovca1* are localized at this region and have been studied in mice (Carter et al., 2000; Toyo-oka et al., 2004; Yu et al., 2014). *Mnt* is essential for embryonic development and survival and may play a role in craniofacial defects associated with MDS, since *Mnt* knockout mice have cleft palate and retardation of skull development (Toyo-oka et al., 2004). *Hic1* knockout mice show no neuronal migration defects or disorganization of the cerebral cortex, but do show gross developmental defects ranging from exencephaly to limb abnormalities (Carter et al., 2000). *Ovca1* encodes a protein involved in diphthamide biosynthesis, which is critical for craniofacial development. *Ovca1* deficiency in tissue derived from the neural crest has been shown to contribute to the craniofacial defects associated with MDS (Yu et al., 2014). Aberrant craniofacial development has been best observed in *Mnt, Hic1*, and *Ovca1* knockout mice (Yu et al., 2014), so the study of the facial dysmorphic features associated with MDS currently relies on these models.

Heterozygous *Mnt*^+/−^, *Hic1*^+/−^, and *Ovca1*^+/−^ mice do not have distinctive developmental phenotypes (Carter et al., 2000; Yu et al., 2014). This raises a question about the representativeness of these models to the human population with MDS, since these individuals only have deletions in one of their chromosome 17p13.3 regions. In most cases, we are currently analyzing craniofacial dysmorphisms resulting from haploinsufficiency of a relatively large region by use of homozygous mouse model knockouts of individual genes. If deletion of these genes results in phenotypes in a dose-dependent manner, developmental phenotypes not seen in the heterozygous single knockout models may emerge when heterozygous knockouts of multiple genes are created. Study of the craniofacial dysmorphisms associated with MDS could be improved by creating *Mnt/Hic1, Hic1/Ovca1*, and/or *Mnt/Ovca1* double heterozygous knockout mice. A *Mnt*/*Hic1*/*Ovca1* triple heterozygous knockout model may show a more dramatic phenotype.

There are *Pafah1b1, Ywhae*, and *Crk* single knockout models and a *Pafah1b1*/*Ywhae* double knockout model available to study the neurodevelopmental defects associated with deletion of chromosome 17p13.3. *Pafah1b1* encodes Lis1, which is involved in neuronal migration and has a role in dendritic filopodia dynamics and spine turnover (Hirotsune et al., 1998; Yamada et al., 2009; Sudarov et al., 2013). *Ywhae* encodes 14-3-3ε and is always deleted in patients with MDS. 14-3-3ε binds Ndel1 (also known as Nudel), which is phosphorylated by CDK5/p35, so Ndel1 remains phosphorylated (Toyo-oka et al., 2003). Single knockout models for *Pafah1b1* and *Ywhae* are associated with similar defects in brain development and neuronal migration, while a double knockout of *Pafah1b1* and *Ywhae* leads to a more severe phenotype that may be attributed to the non-sustained phosphorylation of Ndel1 (Toyo-oka et al., 2003).

The roles of *Crk* and *Crk-*like (*Crkl*) genes have been studied using mouse models. *Crk* encodes a signaling protein that functions downstream of Reelin and has roles in cell proliferation, differentiation, migration, and axonal growth (Huang et al., 2004; Tanaka et al., 2004; Matsuki et al., 2008; Park and Curran, 2008; Barros Fontes et al., 2017). Mutation of only *Crk* or *Crkl* does not compromise Reelin signaling and does not produce any obvious anatomical abnormalities (Park and Curran, 2008). However, double knockout of *Crk* and *Crkl* resulted in mice with grossly abnormal brain appearance (Park and Curran, 2008). This suggests overlapping functions for *Crk* and *Crkl* in Reelin signaling. Deletion of *Crk*, along with *Ywhae*, is thought to contribute to the more severe phenotype seen in MDS, as opposed to ILS (Tenney et al., 2011; Barros Fontes et al., 2017). At this time, *Crk*/*Pafah1b1* and *Crk*/*Ywhae* double heterozygous knockout mice are not available. Creation of a *Crk*/*Pafah1b1* double knockout could help determine if deletion of these genes contributes to the lissencephaly phenotype in a dose-dependent manner, as this seems to be the case when *CRK* and *YWHAE* are deleted together in humans (Tenney et al., 2011; Barros Fontes et al., 2017). There is a need for a *Crk*/*Ywhae* double knockout to be created, since combined deletion of these two genes is clinically important and has yet to be analyzed with a mouse model.

### Limitations to current studies and future directions

Conventional cytogenetic analysis, or karyotyping, is not sufficient to detect microdeletion syndromes. Several case studies have used FISH to identify microdeletions of chromosome 17p13.3 in MDS and ILS patients (Kuwano et al., 1991; Kohler et al., 1995; Pilz et al., 1995; Cho et al., 2009). A relatively new alternative method is mental retardation syndrome multiplex ligation-dependent probe amplification (MRS-MLPA), which is capable of testing for multiple microdeletions at once and is less time consuming and less technically complicated than FISH. Another advantage of MRS-MLPA is its ability to detect smaller deletions that could be missed using FISH (Cho et al., 2009). The importance of this is illustrated by a case involving a MDS patient who had a partial deletion of the *PAFAH1B1* locus (Izumi et al., 2007). A commercially available *PAFAH1B1* FISH probe was used in an attempt to diagnose this patient, but due to the nature of the deletion, the patient's test results were normal (Izumi et al., 2007). The partial deletion was later discovered using a smaller sized FISH probe, drawing attention to the inability of FISH to detect relatively small deletions (Izumi et al., 2007). Other case studies have reported similar incidents (Shimojima et al., 2010). Use of FISH for diagnostic purposes may result in misdiagnosis for individuals with partial deletions of *PAFAH1B1*. Thus, the method that is considered the standard laboratory diagnostic tool for MDS has some shortcomings (Izumi et al., 2007). Unfortunately, the same is true of the mouse models currently being used to study MDS.

To date, there is no murine model for deletion of the complete MDS critical region. The lack of such a model is problematic because single or double knockout mice are not representative of the full range of phenotypes seen in MDS patients, who have relatively large deletions. While it would provide a more clinically relevant model, there are technical challenges in creating a mouse model for deletion of the complete MDS critical region. Later, in the conclusion of this paper, we describe these challenges and propose a strategy to address them.

## Chromosome 17p13.3 duplications

### Introduction

Chromosome 17p13.3 is a region of genomic instability that is prone to submicroscopic rearrangements due to a high density of LCRs (Roos et al., 2009; Capra et al., 2012). These submicroscopic rearrangements can lead to microduplications (Roos et al., 2009; Capra et al., 2012). Duplications that cause 17p13.3 microduplication syndrome have diverse mechanisms, come in various sizes, and include a variety of genes. It follows that these microduplications are associated with a diverse array of phenotypes. The phenotypes generally associated with microduplications of 17p13.3 include developmental and psychomotor delay, behavioral problems and autism spectrum disorder (ASD), structural brain abnormalities and malformations, and distinct physical features (Curry et al., 2013). Microduplications have also been associated with limb malformations and cleft lip and palate (Curry et al., 2013). Microduplications in 17p13.3 occur in the same gene region that when deleted causes MDS, and therefore is sometimes referred to as the MDS critical region. The microduplication minimal region has been defined as a 72kb region exclusively containing gene *YWHAE*, which encodes protein 14-3-3ε and is strongly associated with ASD (Bruno et al., 2010; Curry et al., 2013).

17p13.3 microduplication syndrome diagnosis splits duplications into two categories: class I and class II (Bruno et al., 2010). Class I duplications are categorized as containing the gene *YWHAE*, and not *PAFAH1B1*, which encodes protein LIS1 (Bi et al., 2009). Class I duplications can also involve other genes, such as *CRK*. Patients with class I duplications usually display phenotypes characterized by learning disabilities and ASD (Bi et al., 2009; Curry et al., 2013). These individuals may have congenital defects, macrosomia, dysmorphic facial features, mild to severe developmental delay, and behavioral problems such as increased aggression (Bi et al., 2009; Curry et al., 2013). Class II microduplications are any duplication in chromosome 17p13.3 that contains *PAFAH1B1*. Class II duplications can also involve other genes such as *YWHAE* and *CRK* (Bi et al., 2009; Roos et al., 2009; Bruno et al., 2010). Phenotypes generally associated with class II duplications are mild to severe developmental and/or psychomotor delay, hypotonia, and mild brain malformations including microcephaly (Bi et al., 2009; Bruno et al., 2010). In contrast to infants with class I microduplications, infants with class II microduplications may also have small body size at birth. There are some reports of major internal organ abnormalities, such as structural congenital heart disease, in patients with class II duplications (Bi et al., 2009). Seizures were also noted, but were not common phenotypes among both classes of duplications (Bi et al., 2009; Curry et al., 2013; Gazzellone et al., 2014). Overall, there are overlapping but distinct phenotypes observed in class I and class II microduplication syndrome patients.

Popular techniques for diagnosing and classifying 17p13.3 microduplications in patients are FISH, array comparative genomic hybridization (Array CGH), multiplex ligation-dependent probe amplification (MLPA), RT-PCR analysis, gene expression analysis, and chromosomal microarray (Bi et al., 2009; Roos et al., 2009; Bruno et al., 2010; Luk et al., 2014; Nagata et al., 2014; Petit et al., 2014; Eriksson et al., 2015). Array CGH has been used to detect gain of copy number, whereas FISH has been used to confirm duplications and regions (Bi et al., 2009). Case studies have analyzed the parental genomes of patients with 17p13.3 microduplication syndrome to determine potential inheritance patterns. In case studies where both parents were present and available for sequencing, duplications were largely classified as *de novo*. Only a few reported cases were maternally inherited (Bi et al., 2009; Curry et al., 2013). Simple and complex rearrangements have been observed. Suggested mechanisms for these rearrangements include non-homologous end joining (NHEJ) and replication fork stalling and template switching (Gu et al., 2008; Bi et al., 2009; Bruno et al., 2010). Bruno et al. also identified an individual with a microduplication that seemed to be caused by NAHR due to the observation of breakpoints lying within repetitive elements (Bruno et al., 2010). DNA sequencing of junction points was used to assess these hypothesized mechanisms (Bi et al., 2009; Bruno et al., 2010). It has been suggested that the microduplications present in 17p13.3 microduplication syndrome do not arise from stochastic events, but instead from intrinsic architectural features of the genome (Vissers et al., 2007).

### Phenotypes: developmental/psychomotor delay and behavior problems

All patients present in case studies experienced some form of developmental delay, developmental disorder, cognitive impairment, speech abnormality, and/or behavior problems. Developmental delay is seen in both class I and class II duplications, whereas psychomotor delay is generally a characteristic unique to class II duplications (Bruno et al., 2010). Severity of the delay is highly diverse and variable. Case studies have shown developmental delay to vary greatly both between and within families (Curry et al., 2013). There was no correlation between size and location of duplication and degree of developmental delay or intellectual impairment. Curry et al. observed intellectual disability in 66% of patients who took part in their large-scale case studies (Curry et al., 2013). Cases consisted of individuals ranging from those who completed a high school education, to others who had average IQs accompanied by significant behavioral abnormalities, to those who were severely impaired (Curry et al., 2013). Delayed developmental milestones have been observed across patient populations. In a series of case studies performed by Roos et al. one patient with mild delays was observed to sit independently at 11 months, walk at 27 months, and have a vocabulary containing four words at 24 months (Roos et al., 2009). Other patients showed similar delays, with some more severe than others. Specifically, one more severely delayed patient was assessed at 22 months old and was just beginning to sit independently, had no recorded ability to walk, and had no meaningful vocabulary (Roos et al., 2009). In contrast, Bi et al. reported on one patient whose only impairment was fine motor delays by age 15 (Bi et al., 2009). Curry et al. observed behavioral issues in 100% of cases, but failed to observe a consistent behavioral phenotype for the patient population (Curry et al., 2013). Attention deficit hyperactivity disorder (ADHD) was observed in all individuals with duplications involving *YWHAE* (Bi et al., 2009). Behavioral phenotypes observed across case studies and classes of duplications included: poor social relationships, communication impairment, persistent/repetitive behaviors, attention deficit disorder, obsessive compulsive disorder or tendencies, obsessive food seeking, and mild to significant depression in older patients (Bi et al., 2009; Bruno et al., 2010; Hyon et al., 2011; Curry et al., 2013). As with developmental and psychomotor delay phenotypes, behavioral phenotypes were also variable and spanned duplication classes. Mechanistic etiological studies can help shed light on different microduplication region contributions to different variable delays and behavioral phenotypes.

### Phenotypes: autism spectrum disorder (ASD)

Studies have found rare penetrative genetic copy number variations (CNVs) to account for between 5 and 15% of ASD cases. Copy number variations have been found in 8.6% of children diagnosed with autism, all of which were *de novo* (Gazzellone et al., 2014; Eriksson et al., 2015). It is important to note that <1% of autistic patients studied by Eriksson et al. presented with microduplications in 17p13.3, but when looking specifically at case studies of patients with 17p13.3 microduplication syndrome, ~32% presented with some form of ASD diagnosis or tendencies (Curry et al., 2013; Eriksson et al., 2015). Although this specific duplication is rare in the ASD patient population as a whole, ASD comorbidity is somewhat common in the 17p13.3 microduplication patient population. According to Curry et al., there are one or more autism loci in the 17p13.3 region (Curry et al., 2013). *YWHAE* and *CRK* are seen as candidate genes for ASD that arises in patients with 17p13.3 microduplication syndrome. This is due to the observance of autistic-like behaviors and diagnosis of ASD primarily in patients with class I duplications, and overlap of the same genes being duplicated in multiple patients (Curry et al., 2013).

### Phenotypes: brain abnormalities and characteristic physical features

Brain abnormalities and characteristic physical features vary slightly across classes of microduplications, but are subtle across groups. Brain abnormalities have been assessed using MRI technology on few patients involved in case studies (Bi et al., 2009; Roos et al., 2009; Curry et al., 2013). Brain abnormalities are generally observed in individuals with class II duplications, which result in LIS1 overexpression, but the abnormality phenotype is usually minimal (Bi et al., 2009). One patient in a case study performed by Bi et al. had a triplication of *PAFAH1B1* and showed mild volume loss in the cerebellum, dysgenesis of the corpus callosum, and cerebellar atrophy. The brain also appeared smaller, especially in the occipital cortex (Bi et al., 2009). Another subject with a duplication of *PAFAH1B1* showed mild cerebellar volume loss, thinning of the splenium of the corpus callosum, and a smaller brain, once again most notably in the occipital cortex (Bi et al., 2009). Other case studies performed MRI imaging of six patients with 17p13.3 microduplication syndrome and found that three patients showed variable abnormalities of the corpus callosum and cerebellum, and the remaining three patients had normal MRI scans (Curry et al., 2013). Roos et al. performed case studies of patients with 17p13.3 microduplication syndrome and provided brain imaging data for two patients (Roos et al., 2009). MRI imaging of one patient revealed hypoplasia of the corpus callosum and dilation of lateral ventricles. Thinning of white matter may have occurred. The MRI showed a possible increase of the signal intensity paraventricularly. A small pituitary gland and enlargement of the cisterna magna were also observed (Roos et al., 2009). A brain axial CT scan was performed on one patient at two years old and revealed potential delayed myelination, but in a second axial CT scan at age four myelination appeared normal (Roos et al., 2009). A novel inverted 1.4 Mb microduplication that disrupted *PAFAH1B1* in a patient has been reported (Classen et al., 2013). An MRI was performed on the patient presenting with this duplication and results showed diffuse pachygyria with a moderately thick cortex, smooth white-gray border and no microgyri, and mild-posterior predominate lissencephaly (Classen et al., 2013). Results also showed a thin, short corpus callosum and a mildly thin, flat brainstem (Classen et al., 2013). Brain abnormalities observed in this patient are more characteristic of 17p13.3 microdeletions than microduplications, and authors hypothesized that this phenotype was due to insertion of the 1.4 Mb duplication into intron 1 of *PAFAH1B1*, which disrupted normal splicing and effectively inactivated one copy of *PAFAH1B1* (Classen et al., 2013). One case study observed a patient with a novel class I microduplication with slight brain malformations (Capra et al., 2012). An MRI was performed at 5 years of age and revealed a reduction in the volume and thickness of the isthmus of the cingulate gyrus and the splenium of the corpus callosum (Capra et al., 2012). A dysmorphic aspect of the rostrum of the corpus callosum was also observed (Capra et al., 2012). This is a somewhat rare case in which a patient with a class I microduplication also presented with brain malformations. Patients with class I microduplications usually present with dysmorphic facial features, as opposed to brain malformations. Facial features include asymmetric cranium, flat occipital region, dysmorphic appearance, frontal bossing, low set ears, broad nasal bridge, small nose, and hypertelorism (Bi et al., 2009; Roos et al., 2009; Bruno et al., 2010; Curry et al., 2013). Throughout the rest of the body, other general phenotypes have been observed across duplication classes such as abnormal body proportions, long limbs, and anisomelia (Roos et al., 2009; Bruno et al., 2010).

### Phenotypes: overgrowth and related growth factors

Case studies analyzed by Bi et al. observed macrosomia in all but one patient with *YWHAE* duplication, which contrasts the severe growth restriction observed in individuals with duplications in *PAFAH1B1* (Bi et al., 2009). Patients with class I duplications and macrosomia had duplications expanding into *CRK*, which is known to be involved in regulation of growth and cell differentiation. One case study reports a patient with a 1.58 Mb terminal gain of 17p13.3 (Henry et al., 2016). This gain is a class I duplication including *YWHAE* and *CRK*. The patient showed increased growth factors, pathologic tall stature, and entered puberty at 7 years old. This case study is unique in that it provided a detailed endocrinologic evaluation and reported involvement of the anterior pituitary gland. This suggests a potential hormonal mechanism for overgrowth associated with 17p13.3 microduplication syndrome (Henry et al., 2016). Future case studies of patients with class I microduplications in addition to macrosomia, accelerated growth, and/or elevated growth factors should aim to perform detailed endocrinologic analysis of the patient. This will further elucidate the hypothesized hormonal mechanism and allow for a better understanding of the consequences of *CRK* duplication.

### Phenotypes: limb malformations

The *BHLAH9* gene lies in the 17p13.3 microduplication syndrome region, just outside of the MDS critical region in humans, and has been associated with limb malformations (Nagata et al., 2014). Curry et al. hypothesized that duplication of *BHLAH9* was necessary, but not sufficient, for limb malformation and that a complex mechanism including disruption or separation of nearby regulatory elements underlies the development of split hand/foot malformation with long bone deficiency (SHFLD) (Curry et al., 2013). Class I microduplications have been observed in patients with limb malformations such as SHFLD (Curry et al., 2013). For example, a case study reported a triplication involving *BHLAH9* and a segment of *YWHAE* (Luk et al., 2014). A fetus was observed with bilateral split-hand malformation, and the triplication was maternally inherited. In a study of 51 Japanese families, *BHLAH9* duplications were found to be the largest predictor of a range of different limb malformations (Nagata et al., 2014). A dosage effect was also observed in this sample, with the larger duplication, or in some cases triplication, correlating to more dramatic limb malformations. The limb malformations seen in this cohort were SHFLD, split hand/foot malformation (SHFM), and Gollop-Wolfgang complex (GWC) (Nagata et al., 2014). Like Curry et al., Nagata et al. also suggested that *BHLAH9* copy number gains were necessary, but not sufficient, to cause limb malformations (Curry et al., 2013; Nagata et al., 2014). Case studies on 13 families with both *BHLAH9* copy number gains and limb malformations found hand malformations in 75% of patients, foot malformations in 38% of patients, and long bone deficiency in 43% of patients (Petit et al., 2014). It has been hypothesized that duplications involving a 173 kb critical region correlate with SHFLD, due to the presence of overlapping microduplications in 17p13.3 in three patients involved in a case study (Armour et al., 2011). This 173 kb critical region contains gene *BHLAH9* and exons 1-3 of *ABR*, another gene that lies just outside the MDS critical region (Armour et al., 2011). It has been proposed that duplication of this 173 kb region could potentially alter the dosage of a regulatory element involved in limb development or disrupt the interaction between a nearby regulatory element and its gene target or targets (Armour et al., 2011). These hypothesized mechanisms support the previously suggested mechanism that disruption or separation of nearby regulatory elements from their gene targets in the 17p13.3 microduplication region underlies the development of SHFLD (Curry et al., 2013).

### Phenotypes: cleft lip and palate

Syndromic and non-syndromic cleft lip and palate have been observed in patients with both class I and class II 17p13.3 microduplication syndrome. *YWHAE* has been implicated in the etiology of this phenotype, because it is specifically evidenced in the development of midline craniofacial structures (Tucker and Escobar, 2014). It has been hypothesized that interactions between other genes in the duplication region and *YWHAE* contribute to the variations seen in this phenotype (Tucker and Escobar, 2014). Tucker and Escobar report on two cases of 17p13.3 class I microduplications involving *YWHAE* with cleft lip and palate phenotypes. These cases were the first reported cases with a strictly class I microduplication categorization (Tucker and Escobar, 2014). It was hypothesized that *YWHAE* duplications played a potentially causative role in the cleft lip and palate phenotype (Tucker and Escobar, 2014). A case study also reported a patient with 17p13.3 microduplication syndrome with a non-syndromic cleft lip and palate phenotype (Ibitoye et al., 2015). Curry et al. reported on two families with multiple members who had the 17p13.3 microduplication syndrome and cleft lip and palate with or without accompanying intellectual disability (Curry et al., 2013). Overall, cleft lip and palate is generally accepted as a rare phenotype observed in 17p13.3 microduplication syndrome patients (Curry et al., 2013).

### Limitations to current studies and future directions

The majority of information about 17p13.3 microduplication syndrome has been obtained through case studies in human populations. Because 17p13.3 microduplication syndrome is classified as a rare disease, there are a very limited number of patients available for study. The largest review of case studies was authored by Curry et al. in collaboration with researchers, clinicians, and hospitals across the country to review 21 families (Curry et al., 2013). There are also no studies to date that have analyzed brains post-mortem due to the rarity of the disease and difficulty to obtain samples, so we must rely on techniques such as MRI to observe whole brain malformations. Additionally, gene expression patterns specific to the brain tissue of 17p13.3 microduplication syndrome patients has not been studied due to the limitations of obtaining post-mortem samples. Because participant numbers are small and phenotypes are incredibly variable, it is difficult to find distinct patterns between genotype and phenotype as well as disease etiology. Case studies allow for determining the presence and location of duplications, but difficulties arise when trying to determine mechanistic information.

A transgenic mouse model to conditionally overexpress Lis1 in the developing brain has been created (Bi et al., 2009). This model utilized the Cre-loxP system to increase expression of *Pafah1b1*, which caused a ~20% increase of Lis1 protein levels (Bi et al., 2009). Results showed that increased Lis1 expression in the developing brain may lead to neuronal migration defects as well as smaller brain size (Bi et al., 2009). They observed disorganization in the ventricular zone and disrupted cell polarity, which is critical for migration. Bi et al. also observed both radial and tangential migration disruptions in the cortex and an increase of apoptotic cells (Bi et al., 2009). This Lis1 overexpression mouse model was effective for investigating the effects of Lis1 overexpression in the developing mouse brain. However, in order to create a disease model for the 17p13.3 microduplication syndrome, other genes and proteins must also be considered, as almost all human patients have duplications that span multiple genes.

Cornell et al. used the technique of *in utero* electroporation to observe the effects of 14-3-3ε overexpression in the developing murine cortex (Cornell et al., 2016). Plasmids containing 14-3-3ε overexpression vectors were injected into the lateral ventricle of E14.5 or E16.5 embryonic brains. Electrodes were then positioned to direct plasmids into cells near the ventricular zone, which later migrate to their positions in the cortex. Embryos were then placed back into the uterus and allowed to develop uninterrupted. Brains were harvested and analyzed at P15. Results show that overexpression of 14-3-3ε results in a decrease of neurite formation through interactions with doublecortin (Dcx) (Cornell et al., 2016). This interaction decreases Dcx degradation and results in the failure of microtubule invasion into lamellipodia, which is a key step in neurite formation. Failure of microtubule invasion into the lamellipodia resulted in severe neuronal morphological defects. This was a useful technique for analyzing phenotypic differences in the developing mouse brain between 14-3-3ε overexpressing mice and control mice, but this system does not provide an exact mimicry of 14-3-3ε overexpression in human patients with the 17p13.3 microduplication syndrome. As was the case with Bi et al., this 14-3-3ε overexpression model proved effective for analyzing the effects of a single protein overexpression on neuronal development (Bi et al., 2009; Cornell et al., 2016). However, because both class I and class II duplications involve an interaction between multiple duplicated genes to create a phenotype, a mouse model must be created that encompasses multiple genes in chromosome 17p13.3 that have been identified as critical regions in humans.

Similar difficulties arise when studying deletions in 17p13.3. Models have been created to assess specific genes, but a successful mouse model has not yet been created to observe either 17p13.3 microdeletions or microduplications as they exist in human populations. Future directions include modification of available transgenic technology to create mice expressing certain 17p13.3 microdeletions or microduplications that correlate with specific known human phenotypes for mechanistic and/or etiological studies.

## Mouse model with 17p13.3 deletion or duplication

### Proposed Cre-LoxP and CRISPR/Cas9 combinatorial strategy for creating 17p13.3 deletion or duplication mouse models

To better understand both 17p13.3 microduplications and microdeletions, a new disease model must be created that encompasses critical regions containing both *Pafah1b1* and *Ywhae*. There is a noted interaction between LIS1 and 14-3-3ε in all discussed disorders and both proteins are essential for correct neuronal migration (Toyo-oka et al., 2003; Bi et al., 2009; Bruno et al., 2010). Complications arise when trying to create models containing deletions or duplications of both *Pafah1b1* and *Ywhae* because of the relatively large distance of ~1.3 Mbps between the two genes. This region is too large to either delete or duplicate using traditional Cre-loxP methods or the CRISPR/Cas9 system alone. We propose a methodology to create double transgenic mouse models using both Cre-loxP and CRISPR/Cas9 in combination to delete or duplicate a region of 17p13.3 containing both *Pafah1b1* and *Ywhae*.

**Figure 2:**
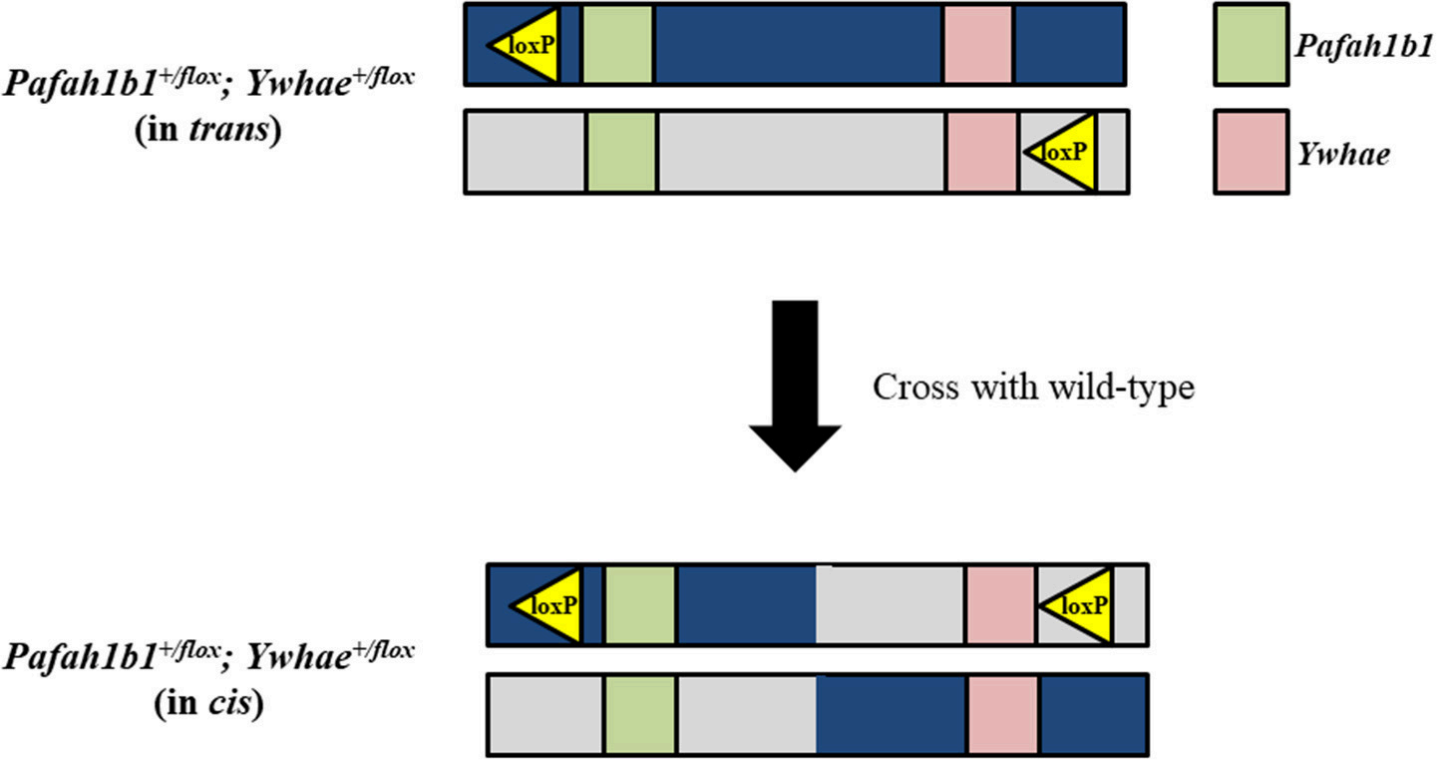
Traditional Cre-loxP approach for generating a mouse model for deletion of the complete MDS critical region. To produce *Pafah1b1*^+/*flox*^*;Ywhae*^+/*flox*^ (in *cis*) mice, and create the opportunity for Cre-mediated deletion to occur, *Pafah1b1*^+/*flox*^*;Ywhae*^+/*flox*^ (in *trans*) mice should be crossed with wild-type mice.

In theory, traditional Cre-loxP methods could be used to make a mouse model for deletion of the complete MDS critical region, but in practice this process would be quite laborious and likely require multiple attempts before mice with the correct genotype could be generated. Mice with loxP sites on the same DNA strand at both *Pafah1b1* and *Ywhae* would need to be produced. A wide range of Cre transgenic mice could then be used to spatially and temporally delete the MDS critical region in mice. It would be possible to produce the *Pafah1b1*^+/*flox*^*;Ywhae*^+/*flox*^ (in *cis*) mice by crossing *Pafah1b1*^+/*flox*^*;Ywhae*^+/*flox*^ (in *trans*) mice with wild-type mice (Figure 2). This approach would rely on a meiotic crossover between alleles to place both loxP sites on the same chromosome (in *cis*). If there is no recombination, offspring would be positive for a loxP site at either the *Pafah1b1* or *Ywhae* alleles. If crossover does occur, then the offspring will be positive for both alleles. The crossover could be confirmed by mating the *Pafah1b1*^+/*flox*^*;Ywhae*^+/*flox*^ (in *cis*) mice to wild-type mice. We would expect ~1% embryonic recombinants from *trans* to *cis* (Merscher et al., 2001). This approach would require genotyping of hundreds of pups and relies heavily on chance, but has been used successfully in the past (Merscher et al., 2001). An additional concern is the Cre-mediated deletion would likely have an extremely low efficiency. The farther apart the loxP sites are, the more inefficient the deletion (Coppoolse et al., 2005).

**Figure 3:**
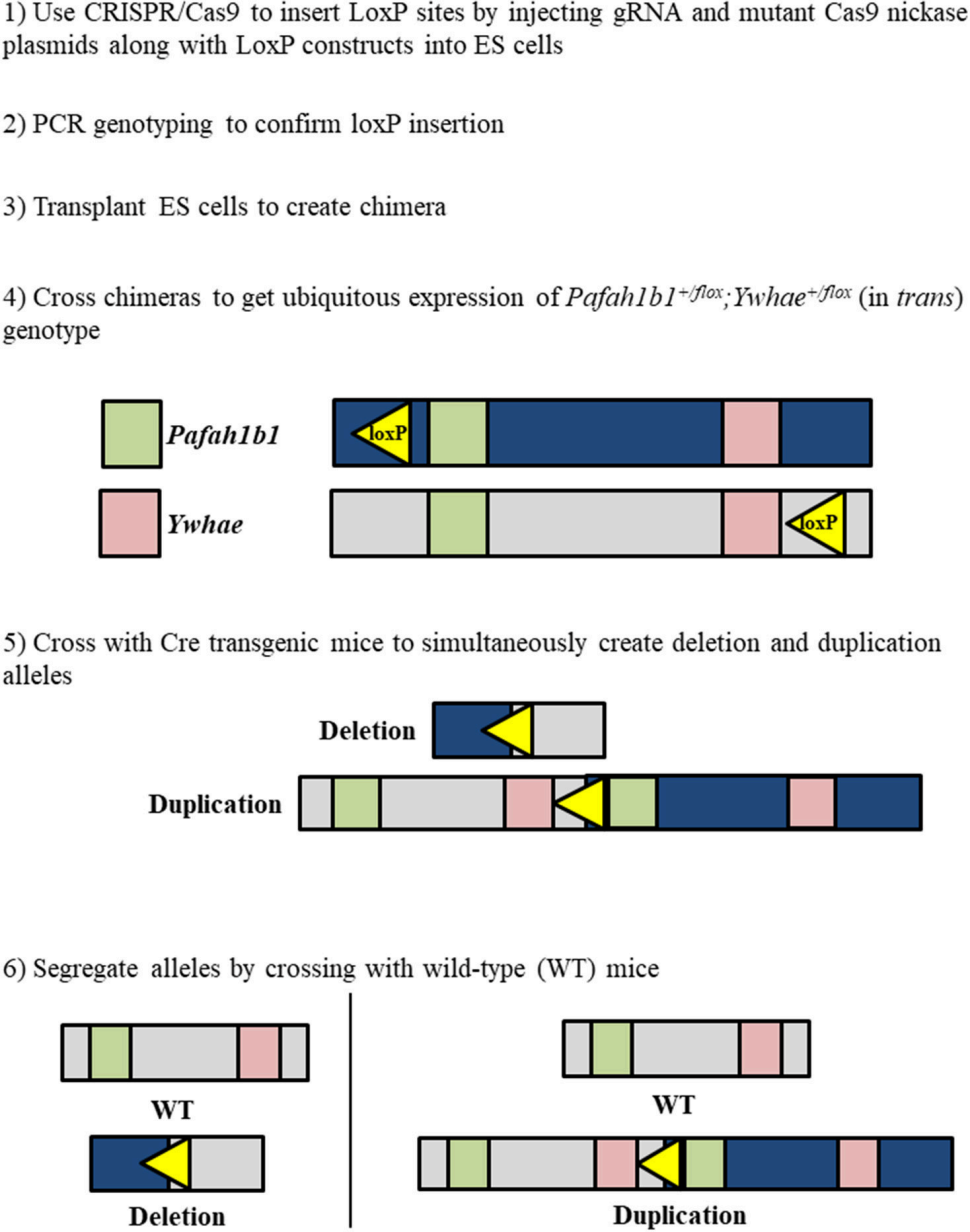
Workflow to simultaneously generate chromosome 17p13.3 deletion and duplication mouse models using a combinatorial CRISPR/Cas9—Cre-loxP approach.

The approach for creating a mouse model for duplication of chromosome 17p13.3 is similar to that for creating the deletion model, with a few crucial differences. To create the duplication, the loxP sites should be on opposite DNA strands (Figure 3). *Pafah1b1*^+/*flox*^*;Ywhae*^+/*flox*^ (in *trans*) mice would be crossed with Cre transgenic mice. This will result in the production of two alleles at the same time. One allele will have a duplication of the MDS critical region, while the other will have a deletion of the MDS critical region. This technique may also be used as an alternative approach for producing the deletion model. The two alleles can be segregated by crossing the mice with the deletion and duplication alleles with wild-type mice. However, a concern is that the recombination efficiency by Cre in *trans* is extremely low (Liu et al., 1998; Zheng et al., 2000). Therefore, this technique is interesting, but requires hard work.

**Figure 4:**
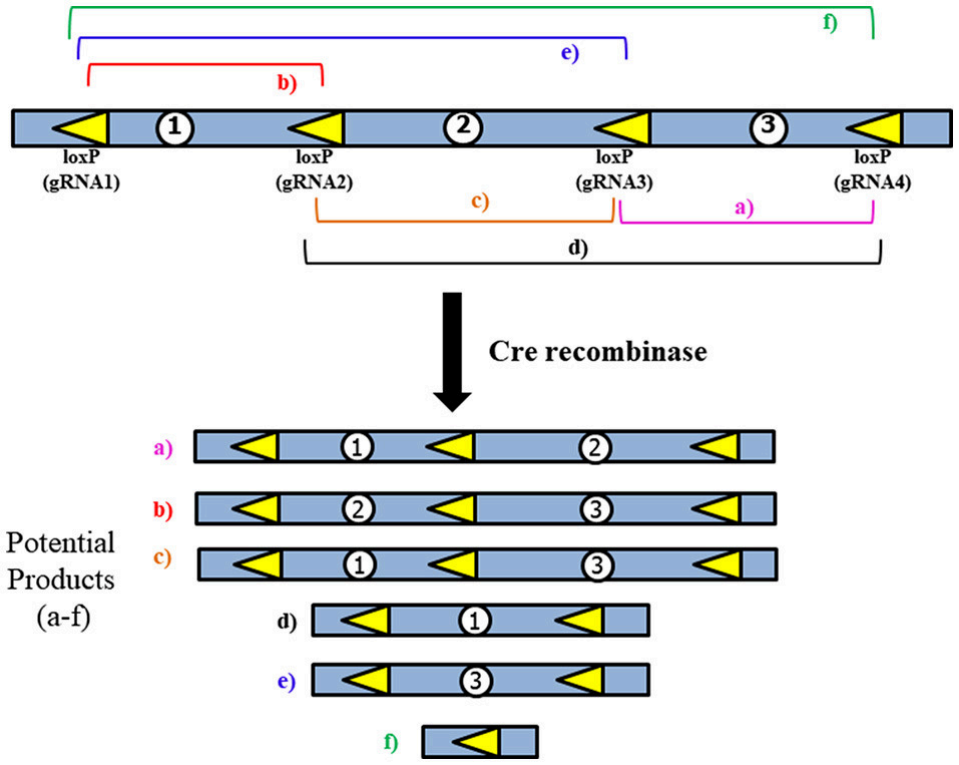
General strategy for use of multiple loxP sites to increase Cre-mediated deletion efficiency. LoxP sites would be inserted using the CRISPR/Cas9 system. The multiple products that could potentially be generated may aid in creating diverse models of multiple types of deletions, which would be similar to what is observed in human patients.

Use of the CRISPR/Cas9 system alone would only allow the deletion of a single gene for each gRNA construct. If two gRNAs were designed to target both *Pafah1b1* and *Ywhae*, mutation would likely occur close to the target sites, leaving most of the genes in the MDS critical region intact. A combined approach using CRISPR/Cas9 to insert the loxP sites near *Pafah1b1* and *Ywhae* would be the most efficient way to create a mouse model with this relatively large deletion (Figure 4). This would allow researchers to bypass the laborious task described above of crossing the *Pafah1b1*^+/*flox*^*;Ywhae*^+/*flox*^ (in *trans*) mice with wild-type mice. The loxP sites inserted by CRISPR/Cas9 should have the same directional orientations, so that the floxed sequence will be deleted. One concern is ensuring that both the loxP sites are inserted in the same chromosome and on the same DNA strand. To partially address this, a mutated nickase version of the Cas9 enzyme could be used. This would create a single-stranded break, as opposed to the double-stranded break created by the wild-type enzyme, which would make it more likely that the loxP sites would be inserted in the same DNA strand to create *cis* mice. Multiple attempts may be necessary before the loxP sites are inserted, since the efficiency of DNA break by the mutated nickase Cas9 is lower than wild-type Cas9.

While the use of the CRISPR/Cas9 system would make insertion of the loxP sites significantly easier, the issue of extremely low Cre-mediated deletion efficiency remains due to the large size of the region we are proposing to delete. To address this, several loxP sites could be inserted to allow for a serial deletion of the complete MDS critical region (Figure 4). This would be done according to the method proposed above, but instead of only inserting loxP sites near *Pafah1b1* and *Ywhae* multiple loxP sites would span the ~1.3 Mbps region. This should increase the efficiency of the Cre-mediated deletion.

Embryonic stem (ES) cells or fertilized eggs may be used to employ our strategy which combines Cre-loxP and CRISPR/Cas9 techniques. Use of ES cells is preferred because it will be simpler to inject the gRNA constructs in cells in culture and confirm that the loxP sites have been incorporated as desired. Once ES cells of the appropriate genotype have been generated, they may be used to create chimera mice. The chimeras should then be used for testing the germline transmission. These mice with the multiple pairs of loxP sites would finally be crossed with Cre transgenic mice. There are a few considerations that should be made when selecting which of the several varieties of Cre recombinase to use. EIIa-Cre causes recombination very early in development (Lakso et al., 1996). This would be most representative of human patients. However, this phenotype may be embryonic lethal. Nestin-Cre is neuron-specific and would cause recombination later in development (Giusti et al., 2014). This would be less representative of what occurs in human development, but may be the better option if inducing recombination at earlier time points proves to be lethal. Although numerous technical aspects would need to be considered throughout the process, generation of mouse models that have either a deletion or duplication of the region of 17p13.3 that contains both *Pafah1b1* and *Ywhae* would likely provide the most clinically relevant models of the associated diseases to date.

### Advantages and disadvantages of the proposed mouse model

Creation of mouse models for deletion and duplication of the MDS critical region will present unique technical challenges. There are many advantages associated with these models, but they are not without some disadvantages. Mouse models are a classical method for the study of neurodevelopmental disorders, since ethical concerns prevent the study of neurodevelopment using human subjects. Post-mortem analysis of brains and other techniques traditionally used to study phenotypes that emerge later in life do not allow developmental defects to be studied in real time, so these methods will not greatly advance our understanding of neurodevelopmental diseases. For these reasons, use of a mouse model that allows for the observation of neurodevelopment is highly advantageous. With recent advances in the use of iPSCs and organoids, it is possible to observe and analyze cellular, molecular, and simple morphogenic and migrational phenotypes associated with disease. Use of a mouse model would also allow for observation of these phenomena, but would additionally allow for analysis of accompanying behavioral phenotypes and craniofacial defects, which are homologous to those seen in human patients.

Although mice and humans differ in brain structure, chromosome structure, and behavior, creation of a mouse model is still useful to draw conclusions about brain and behavioral abnormalities in humans (Lui et al., 2011; Watson and Platt, 2012; Geschwind and Rakic, 2013; Florio and Huttner, 2014). There is a slight variation in the arrangement of genes surrounding the MDS critical region. The *BHLHA9* gene that is located immediately outside the MDS critical region in humans is separated from the MDS critical region in mice by several other genes. This means that a frequently duplicated gene in humans may not factor into the mouse model for the 17p13.3 microduplication syndrome. Although this slight difference may cause variation, the entire MDS critical region does exist in the same order in humans and mice.

The most relevant behavioral difference between mice and humans is that it is impossible to draw conclusions regarding language development in mice, which would be of interest since language development is delayed in autistic human patients with 17p13.3 microduplication syndrome and developmentally delayed patients with 17p13.3 microdeletion syndrome (Silverman et al., 2010; Watson and Platt, 2012). However, there are other established measures of autistic behavior in mice that are homologous to humans such as stereotypic and repetitive behaviors, social behavior, and social communications (Crawley, 2004; Silverman et al., 2010; Watson and Platt, 2012). Social behavior is an especially important consideration for the study of ASD and related phenotypes in which patients may have characteristically poor social relationships.

Mouse models are also useful for analyzing typical phenotypes observed in patients with 17p13.3 microdeletion syndrome such as spasticity, epileptic seizures, decreased longevity, and intellectual disability (Dobyns et al., 1993). In addition, the craniofacial defects observed in human populations with 17p13.3 microdeletion syndrome are manifested in mice (Carter et al., 2000; Toyo-oka et al., 2004; Yu et al., 2014). Use of a mouse model with a large deletion or duplication, as opposed to single gene deletions and duplications, will be much more representative of the genotypes that occur in patients with ILS, MDS, and 17p13.3 microduplication syndrome. While there are drawbacks associated with any animal model, the creation of the proposed mouse models would advance the study of the rare neurodevelopmental diseases discussed in this review and is a goal worthy of effort.

### Next generation sequencing and rare disease

Although the incidence of each individual rare disease is low, rare diseases collectively affect ~30 million people in the United States (Shen et al., 2015). Next generation sequencing (NGS) has accelerated the rate at which genes responsible for rare monogenic diseases are being identified (Boycott et al., 2013). Whole-exome sequencing (WES) is a popular technique that is currently favored over whole-genome sequencing (WGS) due to its lower cost and complexity for detecting genetic variations (Boycott et al., 2013). WES is an NGS technique that may be applied to identify novel rare disease causing genes. NGS techniques are not yet being used as a typical method for diagnosis, but this may become a common practice once more gene-phenotype relationships are identified (Shen et al., 2015). NGS has had a significant positive impact on rare disease research. However, this impact is rather limited to diseases that are caused by a single gene.

Progress in research has not been as rapid on rare diseases that involve large regions of the genome and multiple genes, such as MDS and 17p13.3 microduplication syndrome, due to the focus of previous research on identifying mutations in single genes. The NGS techniques that have been used to identify about half of the genes responsible for the approximated 7,000 known rare monogenic diseases need to be modified before they may be applied to the study of rare diseases involving multiple genes (Boycott et al., 2013). Specifically, data interpretation should be modified. To help identify the specific genes that have been deleted or duplicated in patients with MDS or 17p13.3 microduplication syndrome, respectively, studies should be looking at CNVs of genes within critical regions for these diseases. Such an approach may help identify additional genes of importance for these conditions. Applying this strategy to other diseases of unknown genetic origin may help determine if multiple genes are deleted or duplicated in other syndromes.

Study of CNVs using targeted NGS sequencing data, such as WES, is challenging because deletions and duplications may not begin or end within the exome, making them difficult to identify. However, a statistical method known as SeqCNV has recently been developed that can robustly identify CNVs using capture NGS data (Chen et al., 2017). First, a dataset is generated using WES. Then, the analysis is done using SeqCNV to identify the copy number ratio and CNV boundary through use of read depth information and maximum penalized likelihood estimation (MPLE) (Chen et al., 2017). CNVs can also be identified from targeted NGS data using a popular software package called ExomeDepth. The sensitivity and specificity of ExomeDepth v1.1.16 were determined to be 100% and 99.8%, respectively, validating this technique as an appropriate method for detection of CNVs (Ellingford et al., 2017). Methods such as SeqCNV and ExomeDepth are also advantageous because they can identify CNVs anywhere in the genome by using WES data, unlike other methods, such as FISH, where a region of interest must be identified to carry out the technique. Development of NGS techniques has already greatly improved our knowledge of rare monogenic diseases. A shift in attention to more complex genotypes involving the deletion or duplication of multiple genes may result in better approaches for identifying CNVs.

## Conclusions

Microdeletions and microduplications of chromosome 17p13.3 lead to rare and complex diseases. The advent of NGS techniques and refinement of CRISPR/Cas9 mouse genetics opens new possibilities for the study of rare diseases such as the creation of new mouse models that previously would have been difficult or impossible to create, advanced analysis of WES data to better identify CNVs, and the potential for more accurate diagnostic tools. These techniques also offer alternate tools to study a rare disease that does not heavily rely on case study data, which is difficult to obtain. Study of neurodevelopmental disorders can also advance the field of neurodevelopmental research in general by contributing to our knowledge about fundamental processes involved in normal brain development including neurogenesis, neuronal migration, and neurite formation.

## Author contributions

SMB, SAB, and TS wrote the initial draft of the manuscript. SMB and SAB: created the figures. KT edited and finalized it.

### Conflict of interest statement

The authors declare that the research was conducted in the absence of any commercial or financial relationships that could be construed as a potential conflict of interest.
